# Supplementary figures and images for: Cell Type-Specific Hormonal Signaling Configures Hypothalamic Circuits for Parenting
Source: bioRxiv. 2025 Dec 12:2025.12.11.693766. Preprint. [Version 1] doi: 10.64898/2025.12.11.693766 (PMC12713651; doi:10.64898/2025.12.11.693766)

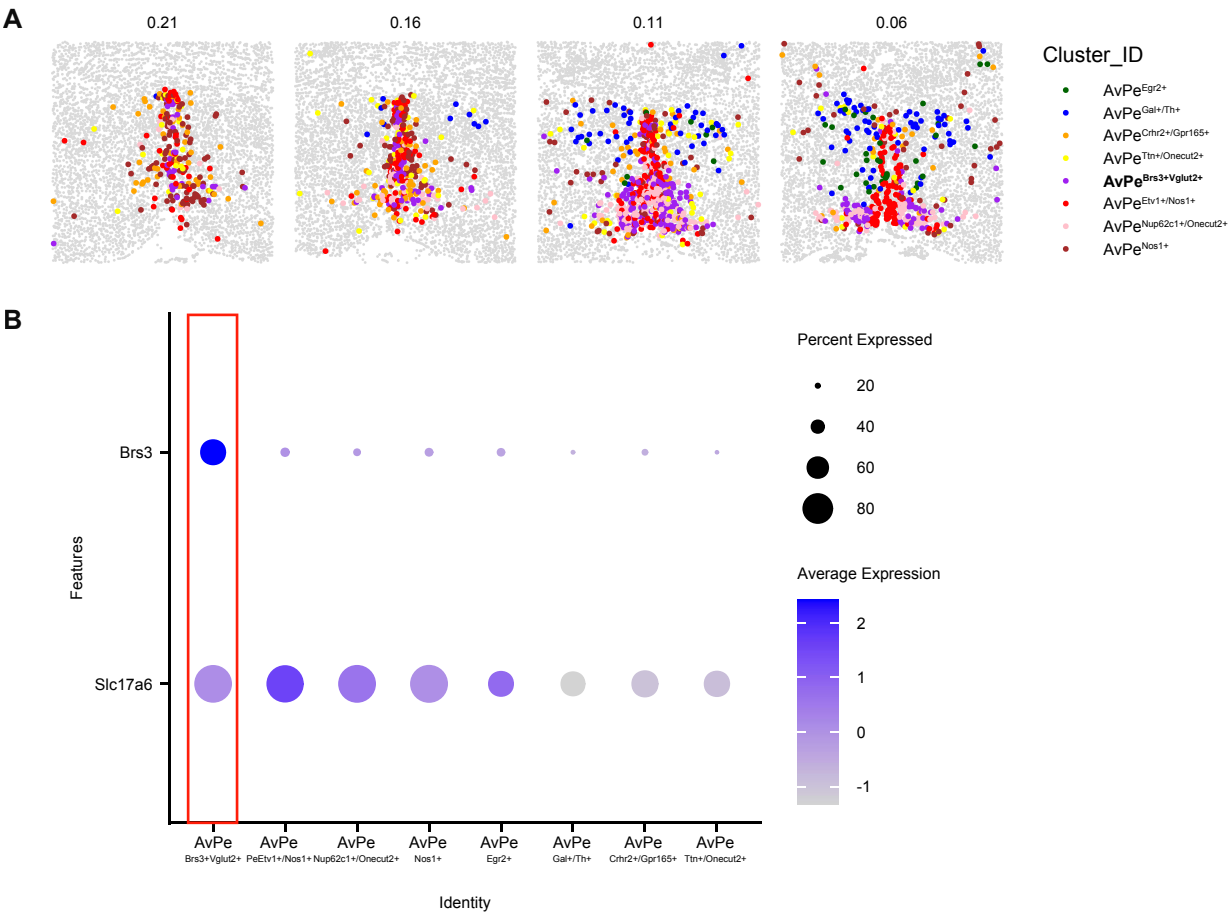

A

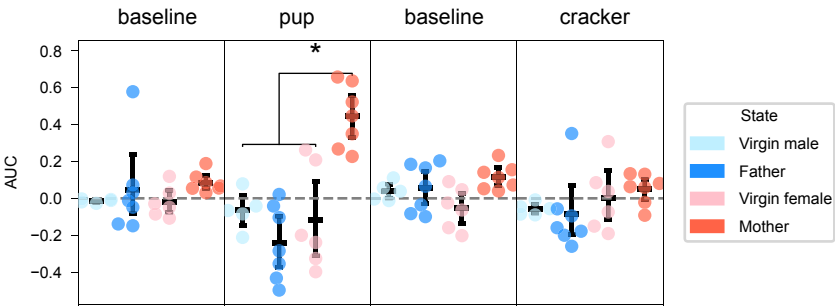

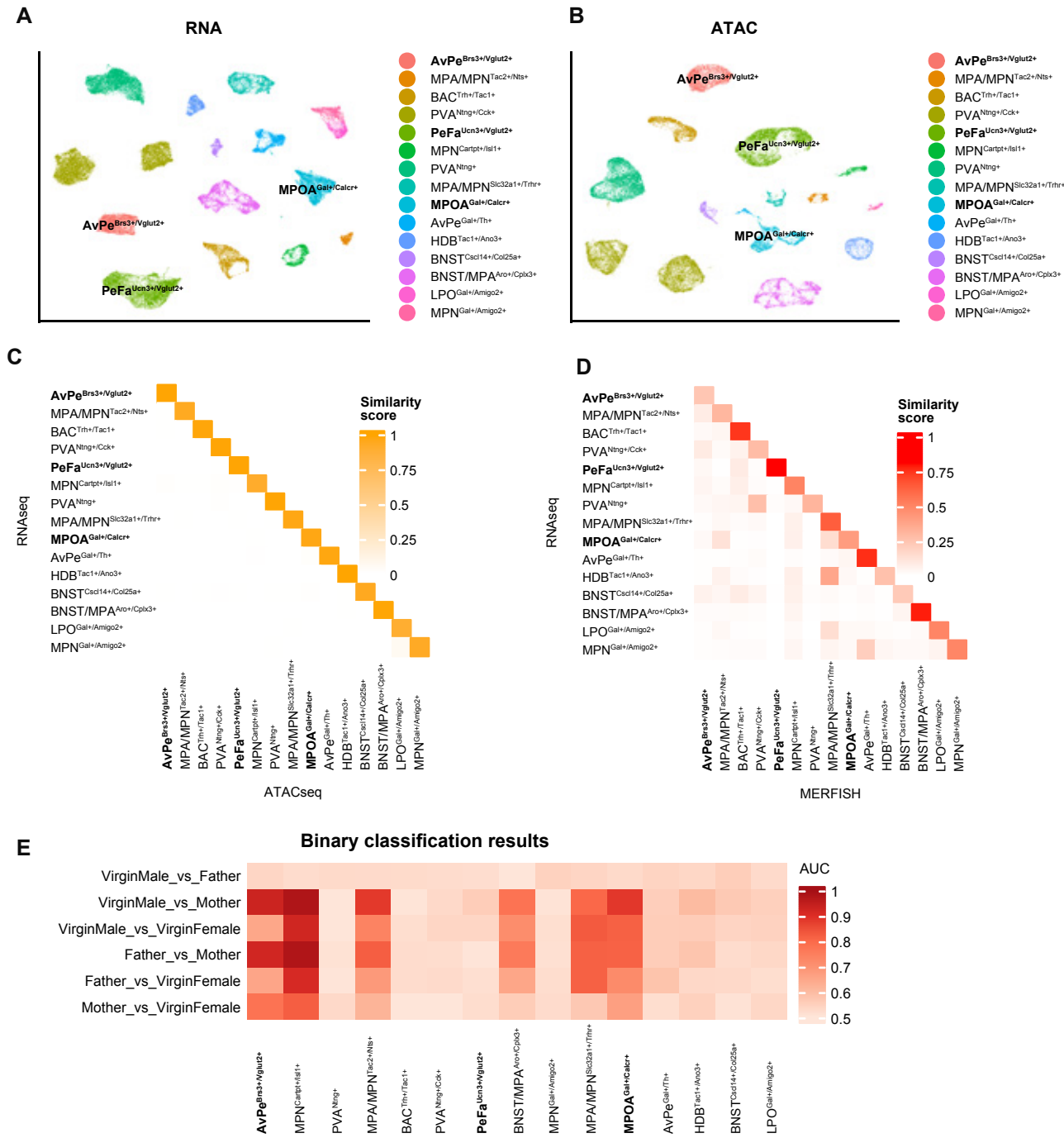

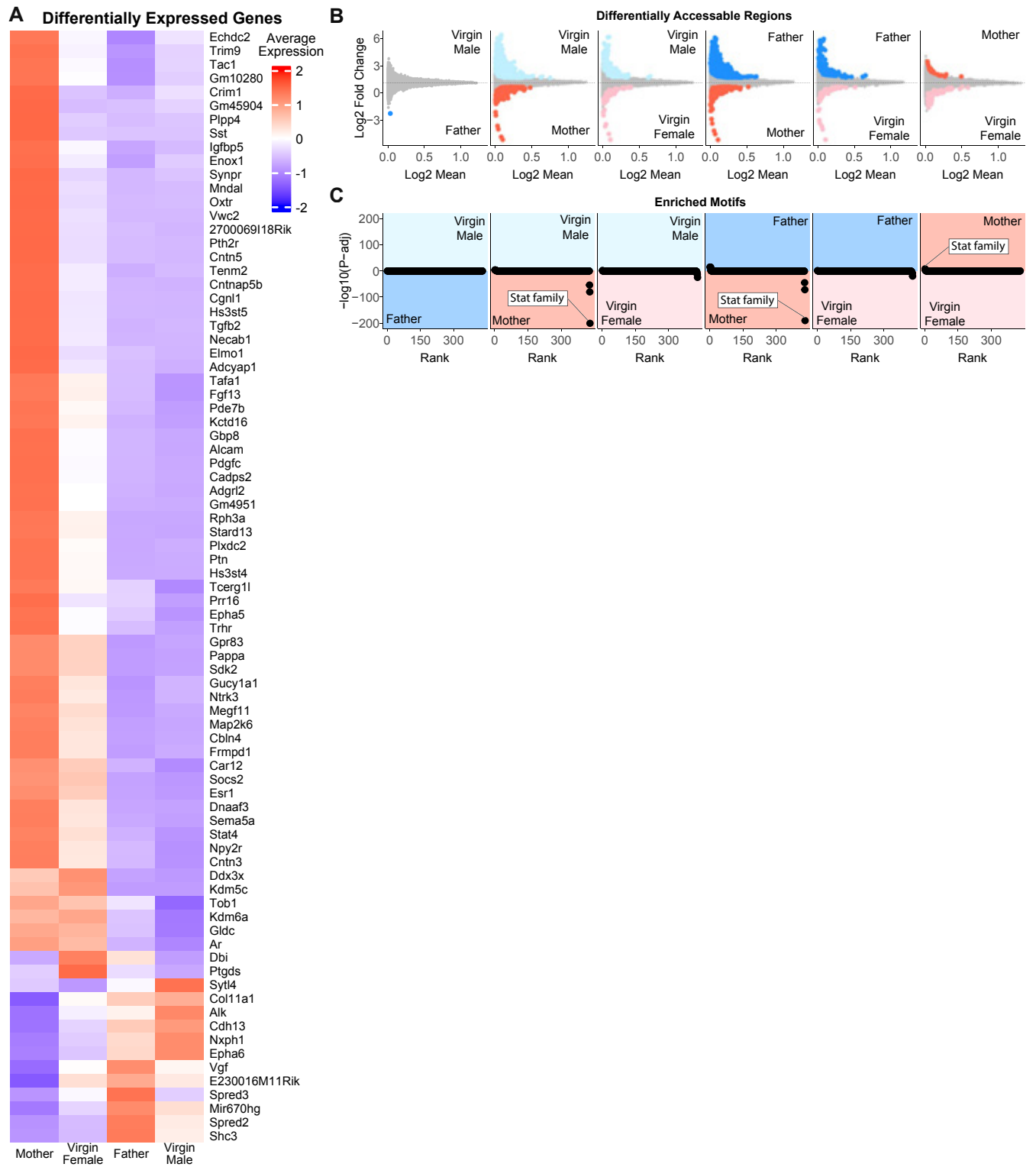

A

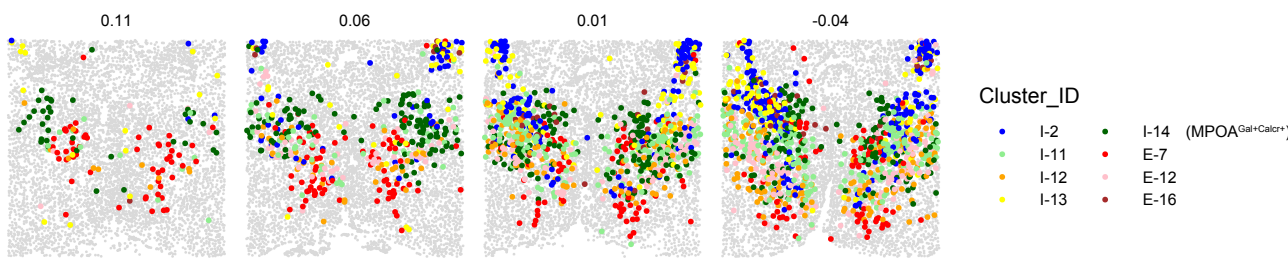

B

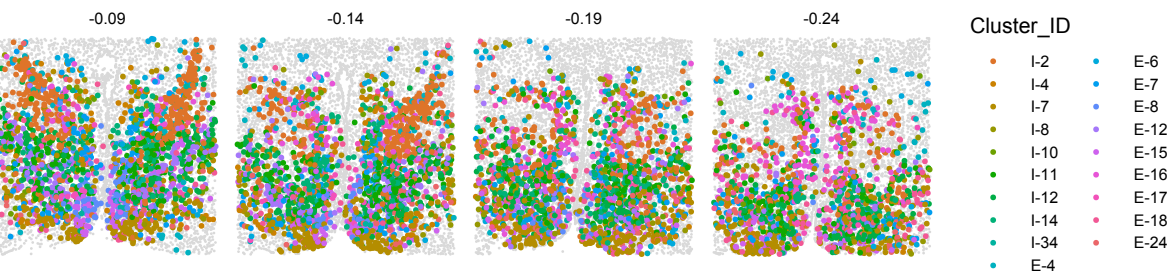

C

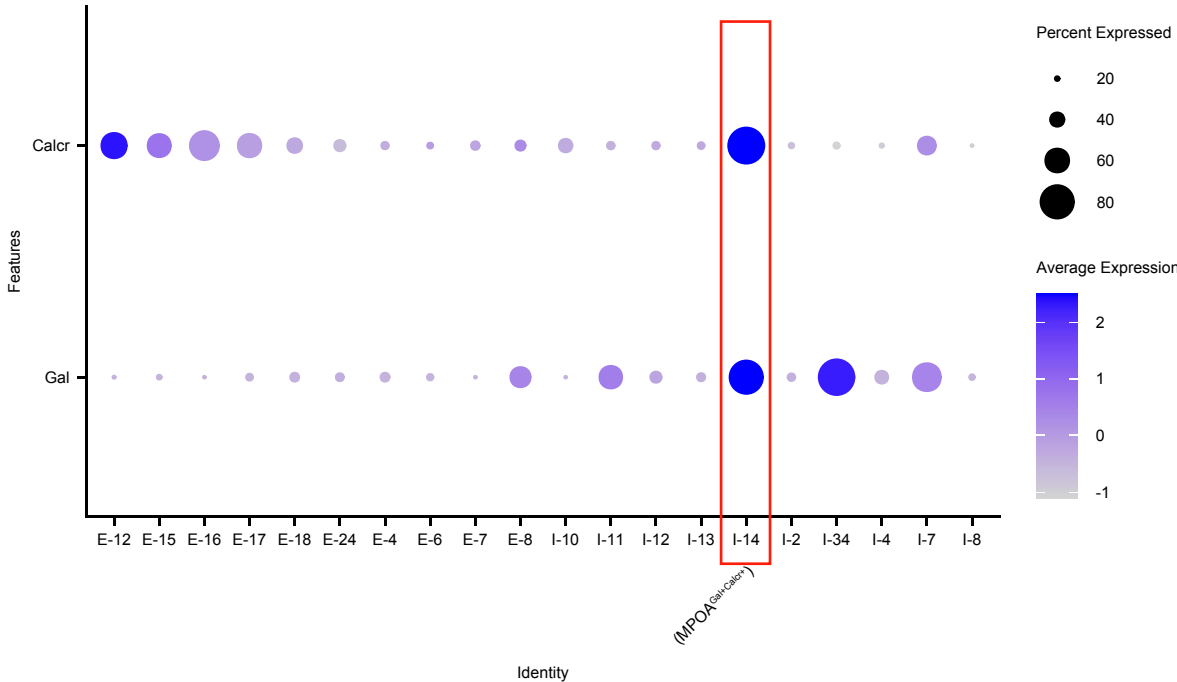

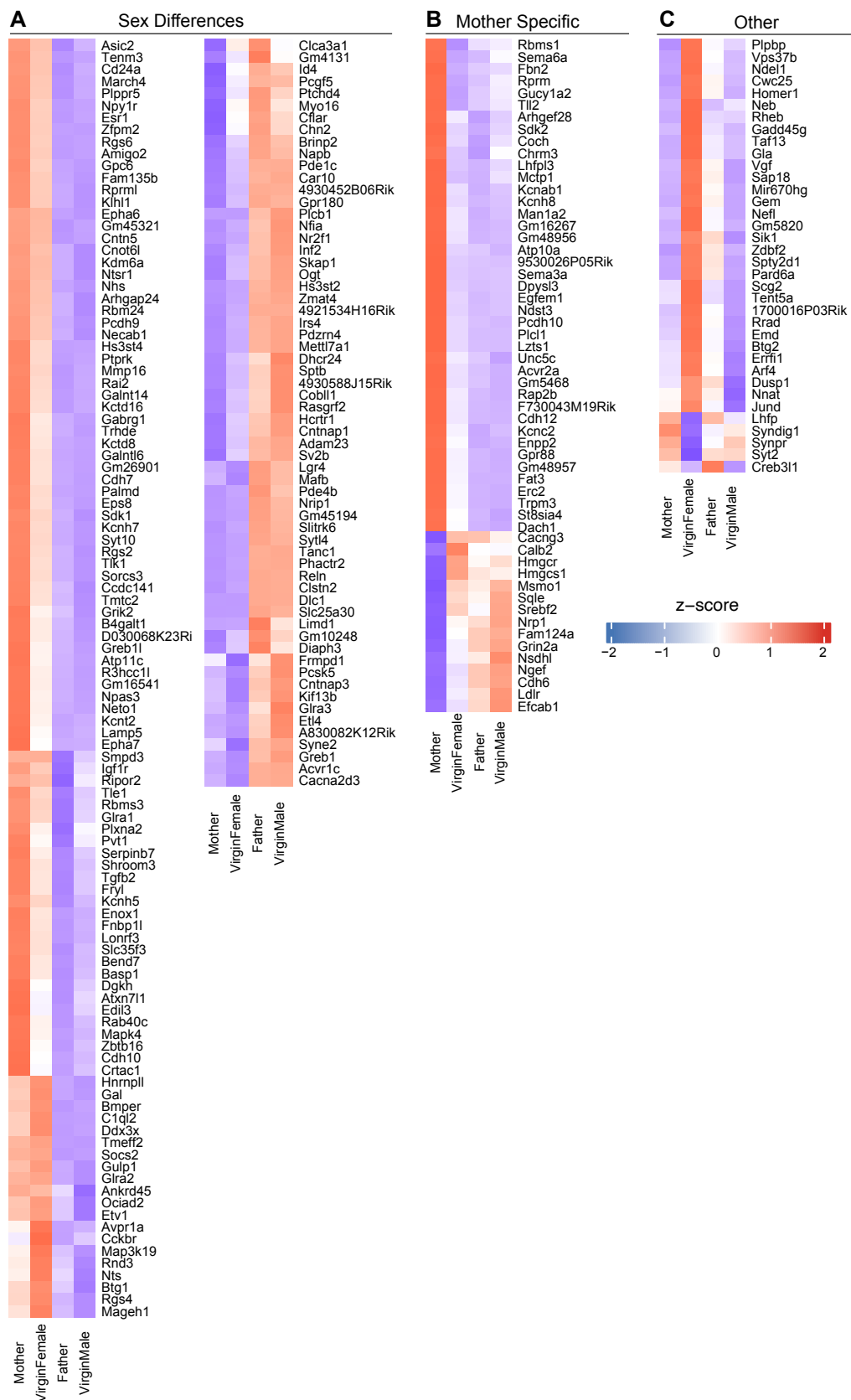

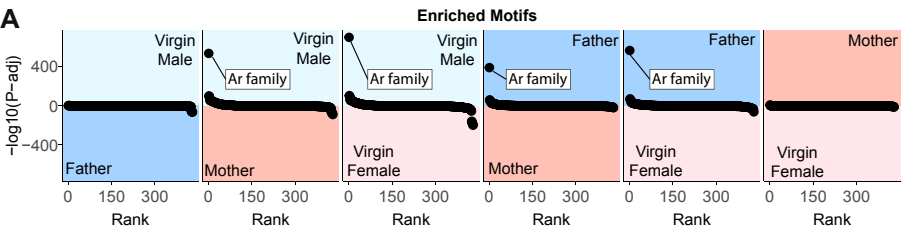

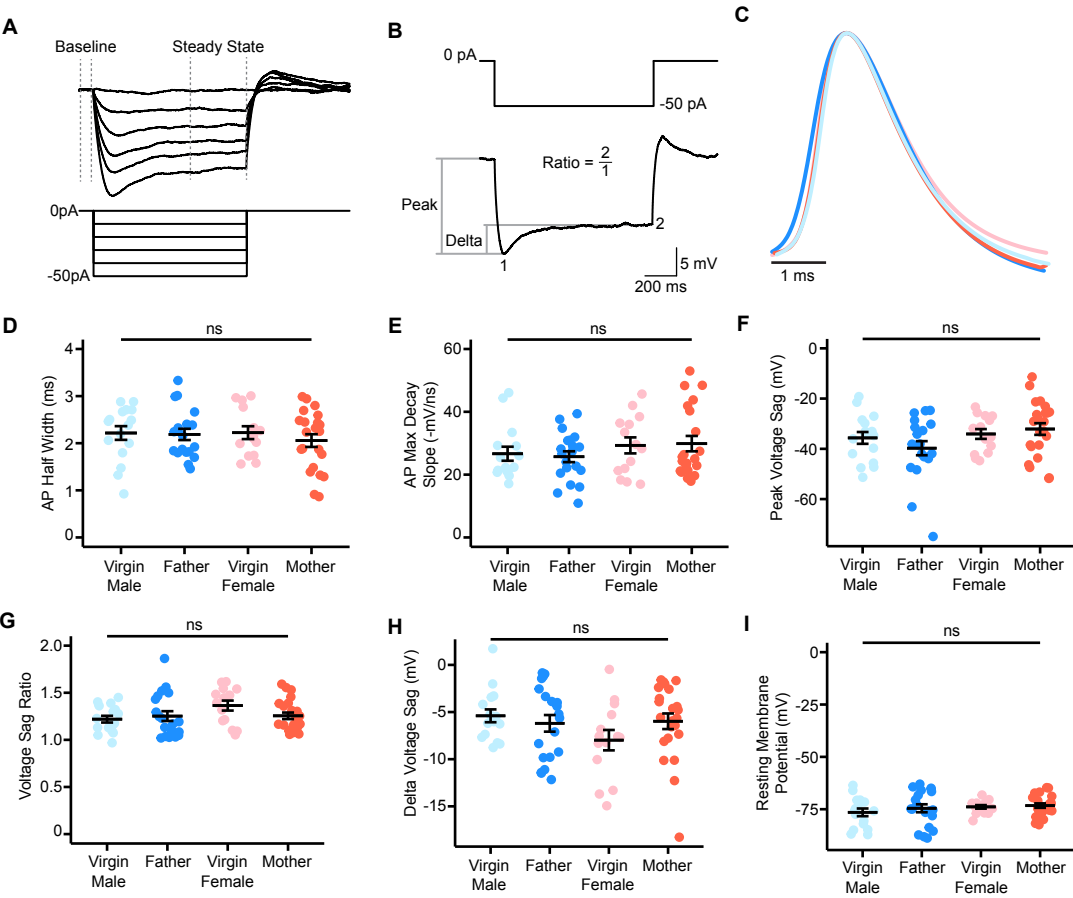

Supplement: 1 — Fig. S1. Brs3 and Slc17a6 serve as molecular markers for AvPeBrs3+Vglut2+ neurons (A) MERFISH images of molecularly defined AvPe cell types (9)(B) Gene expression of AvPe cell types indicating expression profiles that are present in the desired population. Fig. S2. AvPeBrs3+Vglut2+ neurons are active during pup exposure. (A) Cumulative area under the curve of GCaMP7s activity during the duration of assay exposure for all animals. Fig. S3. RNA- and ATAC-seq data map to previous cell types and reveal unique expression patterns (A-B) UMAP plot highlighting the distinct nature of the identified cell types for both molecular modalities. (C) Canonical correlation score calculated from each cluster compared to all others for RNA- and ATAC-seq modalities. (D) Same as in (C) but comparing the RNA-seq dataset to the previously published MERFISH dataset from Moffitt et al. (E) Area under the curve score for cell type-specific random forest classification results of all pair-wise decoding combinations. Fig. S4. AvPeBrs3+Vglut2+ neurons show unique molecular profile in mothers (A) All 81 differentially expressed genes between animal sex and state hierarchically clustered. (B) Pair-wise log2Fold change plots of differentially accessible regions for all combinations of samples. Color represents significant differences in accessibility. (C) Pair-wise −log10 plots of transcription factor motifs enriched in accessible peaks for all combinations of samples. Fig. S5. Gal and Calcr serve as molecular markers for MPOAGal+Calcr+ neurons (A) MERFISH images of defined cell types from Moffit et al located in the StHy. (B) MERFISH images of defined cell types from Moffit et al located in the MPN. (C) Gene expression of StHy and MPN localized cell types indicating expression profiles that are present in the desired population. Fig. S6. MPOAGal+Calcr+ neurons show complex differences in gene expression patterns (A) Genes that show sex differences in expression. (B) Genes showing expression chan [file NIHPP2025.12.11.693766V1-supplement-1.pdf]
